# Ageing-Induced Decline in Primary Myeloid Cell Phagocytosis Is Unaffected by Optineurin Insufficiency

**DOI:** 10.3390/biology12020240

**Published:** 2023-02-03

**Authors:** Josip Peradinovic, Nikolina Mohovic, Katarina Bulic, Andrea Markovinovic, Raffaello Cimbro, Ivana Munitic

**Affiliations:** 1 Laboratory for Molecular Immunology, Department of Biotechnology, University of Rijeka, R. Matejcic 2, 51000 Rijeka, Croatia; 2 Department of Basic and Clinical Neuroscience, Maurice Wohl Clinical Neuroscience Institute, Institute of Psychiatry, Psychology and Neuroscience, King’s College London, London SE5 9RT, UK

**Keywords:** phagocytosis, ageing, optineurin, neurodegeneration, inflammation

## Abstract

**Simple Summary:**

Amyotrophic lateral sclerosis (ALS) is a devastating disease, which results in death as early as 2–5 years upon diagnosis. Finding a cure for ALS has proved to be extremely challenging due to its complex genetic background, and its still largely unknown environmental triggers. A recent study has pinpointed inefficient phagocytosis as a new common disease mechanism in patients of different genetic backgrounds. Here, we analysed if phagocytosis defects are directly linked to mutations in the *OPTN* gene, which encodes for optineurin protein that regulates inflammatory signalling. To this end, we used innate immune cells (macrophages and microglia) from an optineurin truncation mouse model that resembles some patient mutations. We analysed these cells at steady state and stressed by the common ALS risk factors—ageing and inflammation. We observed that ageing decreased phagocytosis, whereas inflammation decreased degradation of the phagocytosed material. However, none of these processes were affected by the *OPTN* mutation. This suggests that the disease mechanism in patients carrying *OPTN* mutations is distinct.

**Abstract:**

Optineurin is a ubiquitin-binding adaptor protein involved in multiple cellular processes, including innate inflammatory signalling. Mutations in optineurin were found in amyotrophic lateral sclerosis, an adult-onset fatal neurodegenerative disease that targets motor neurons. Neurodegeneration results in generation of neuronal debris, which is primarily cleared by myeloid cells. To assess the role of optineurin in phagocytosis, we performed a flow cytometry-based phagocytic assay of apoptotic neuronal debris and *E. coli* bioparticles in bone marrow-derived macrophages (BMDMs), and primary neonatal microglia from wild-type (WT) and optineurin-insufficient (Optn^470T^) mice. We found no difference in phagocytosis efficiency and the accompanying cytokine secretion in WT and Optn^470T^ BMDMs and microglia. This was true at both steady state and upon proinflammatory polarization with lipopolysaccharide. When we analysed the effect of ageing as a major risk factor for neurodegeneration, we found a substantial decrease in the percentage of phagocytic cells and proinflammatory cytokine secretion in BMDMs from 2-year-old mice. However, this ageing-induced phagocytic decline was unaffected by optineurin insufficiency. All together, these results indicate that ageing is the factor that perturbs normal phagocytosis and proinflammatory cytokine secretion, but that optineurin is dispensable for these processes.

## 1. Introduction

Amyotrophic lateral sclerosis (ALS) is the fastest progressing adult motor neuron disease and has no cure [1,2]. Patients with family history comprise 10% of ALS cases (familial ALS or fALS), while the remaining 90% are sporadic (sALS), often linked to de novo and/or incomplete penetrance mutations in the same genes mutated in fALS patients. Most frequent mutations are found in chromosome 9 open reading frame 72 (*C9ORF72*), superoxide dismutase 1 (*SOD1*), *TARDBP* encoding for TAR DNA-binding protein of 43 kDa (*TDP-43*), and fused in sarcoma (*FUS*). However, more than 50 genes have been reported to cause or modify ALS [3,4,5]. They encode for a heterogenous set of proteins, which can lead to increased protein aggregation, impaired RNA metabolism, dysfunctional nucleocytoplasmic, vesicular and axonal trafficking, oxidative stress, excitotoxicity, and mitochondrial dysfunction. The intriguing finding that dysregulation in so many different genes and molecular pathways can converge and trigger motor neuron death has been extensively reviewed [6,7,8], but is still unclear if, how, and when during ALS progression they crosstalk [9,10]. Notably, the immune system dysregulation accompanies all ALS pathologies, regardless of genetic background [11,12]. This dysregulation is almost invariably thought to occur secondary to neuronal damage, rather than function as a primary disease trigger. However, several ALS-linked genes that encode for proteins that directly affect immune cell functions, including TANK-binding kinase (*TBK1*), cylindromatosis (*CYLD*), and optineurin, could also potentially act as primary triggers for immune system dysregulation in ALS [13,14,15,16].

Optineurin is a ubiquitin-binding adaptor protein with a preference for M1 and K63-linked polyubiquitin chains [17]. It has been implicated in various cellular processes, including inflammatory signalling, vesicle trafficking, autophagy, Golgi maintenance, and cell death [18]. The *OPTN* gene mutations in ALS are thought to act by loss-of-function, but the details on how optineurin protects from neurodegeneration are still unclear. For this reason, several mouse models were designed to study the effect of optineurin deficiency or insufficiency. Notably, unmanipulated *Optn*^-/-^ mice do not develop neurodegeneration, evident as lack of neuronal loss and microgliosis [19,20,21]. Mild microglial activation and demyelination with minor motor problems (solely in vertical rearing activity) have been described in one report [20], although this has been challenged by a follow-up study [22]. However, diminished TBK1 activation and IFN-β production upon Toll-like receptor (TLR) stimulation have been reported in macrophages derived from *Optn*^-/-^ mice [19]. Similarly, we have reported diminished TBK1/IFN-β pathway activation in both macrophages and microglia derived from an optineurin insufficiency mouse model (Optn^470T^), which does not bind ubiquitin due to a C-terminal truncation [23,24,25], thus mirroring some of the loss-off-function optineurin truncations found in ALS patients [13,26]. Therefore, optineurin could be neuroprotective by regulating (neuro)inflammation and/or other innate cell functions. 

Ageing is the most important environmental risk factor for neurodegenerative disease. Aged organisms exhibit increased propensity for secretion of proinflammatory factors from innate immune cells, triggering chronic low-grade inflammation, which is referred to as inflammageing [27]. Monocytes, macrophages, and tissue-resident macrophage subsets, including microglia, which represent the only innate immune cells in the CNS parenchyma, are the key innate cells driving the inflammageing [28]. These myeloid subsets are crucial for maintenance of tissue homeostasis in a healthy setting, but also represent the first responders at the times of various acute or chronic insults. Microglia, which are originally yolk-sac derived and cannot arise from circulating monocytes, comprise some of the longest-living self-renewing cells in the human body [29]. It is thus not surprising that they have been shown to degenerate and become less functional with age [30]. Clean-up of protein aggregates, pathogens, and dead cell debris occurs via the process of phagocytosis, which comprises engulfing extracellular particles larger than 0.5 μm into phagocytic cells, fusion of phagosomes with lysosomes and subsequent degradation of phagocytosed material within the lysosomes by low pH, enzymatic lysis or oxidative stress [28,31]. Multiple studies have implicated phagocytosis in the process of neurodegeneration. In pre-symptomatic mutant SOD1 transgenic rat ALS model, phagocytic microglia can be detected adjacent to motor neurons in the lumbar spinal cord before neuronal loss [32]. Microglia associated with neurodegenerative lesions, labelled disease-associated microglia (DAM), have recently been extensively profiled at single-cell level: DAM present in AD and ALS lesions shut down homeostatic genes and upregulate phagocytic, lysosomal, and immune response regulating genes [33]. A recent study has confirmed this in a human model: monocytes from sALS patients differentiated into microglia-like cells showed impaired phagocytosis, which correlated with the patients’ rate of disease progression [34]. 

To study if phagocytosis defects are associated with optineurin insufficiency, we analysed phagocytosis in primary myeloid cells (BMDMs and microglia) from Optn^470T^ mouse model. To this end we set up a flow cytometry-based phagocytosis assay using apoptotic mouse neuroblastoma-derived Neuro2A (N2A) cells as targets. Furthermore, since efficient phagocytic removal of apoptotic cells suppresses proinflammatory and stimulates anti-inflammatory factor production, thereby stimulating healing instead of chronic inflammation and/or auto-immunity [35], we used additional stimuli (lipopolysaccharide and *E. coli* bioparticles) to elicit a proinflammatory environment. This is expected to better mimic the chronic stress that accompanies neurodegeneration. Finally, to analyse the effect of age on phagocytosis, we compared BMDMs from young 3-month-old and 2-year-old mice. 

## 2. Materials and Methods

### 2.1. Reagents

Bafilomycin A1 (#B1793), Cytochalasin D (#C8273), DNAse (#DN25), 4′,6-diamidino-2-phenylindole (DAPI, #D9542), poly-L-lysine (#P1274), lipopolysaccharide from *E. coli* O111:B4 (LPS, #L4391), PKH26 (#PKH26GL), and H_3_PO_4_ (#30417) were from Sigma-Aldrich (St. Louis, MO, USA). Sodium azide (#K305.1) was purchased from Carl Roth (Oberuzwil, Switzerland). Capture antibody against IFN-β (#sc-17563) and staurosporine (#sc-3510) were from Santa Cruz (Dallas, TX, USA). Detection antibody against IFN-β (#32400-1) was from PBL (Piscataway, NJ, USA) and HRP-conjugated goat anti-rabbit IgG (H+L) antibody (#111-035-144) was from Jackson ImmunoResearch (Ely, UK). CellTrace Far Red (#C34572), pHrodo™ Green *E. coli* bioparticles Conjugate for Phagocytosis (#P35366; hereafter referred as *E. coli* bioparticles), Mouse TNF-alpha Uncoated ELISA Kit (#88-7324-88), goat anti-rabbit Alexa Fluor 488 (#A11034), and goat anti-rat Alexa Fluor 546 (#A11081) secondary antibodies were from Invitrogen (Carlsbad, CA, USA). Carboxyfluorescein succinimidyl ester (CFSE, #65-0850-84) was from eBioscience. Anti-Iba1 (#019-19741) was purchased from Wako (Osaka, Japan), and anti-CD68 (#ab53444) from Abcam (Cambridge, UK). LabTek Chamber Slide (#177402) was from Thermo Fisher Scientific (Waltham, MA, USA), Aqua-Poly/Mount mounting medium (#18606-20) from Polysciences, Inc (Hirschberg a der Bergstrasse, Germany), Mouse IL-10 DuoSet ELISA (#RD-DY417) from R&D systems (Minneapolis, MN, USA) and 3,3′,5,5′-Tetrametilbenzidine (TMB, #ES001) from Merck Millipore (Burlington, MA, USA). 4-(2-hydroxyethyl)-1-piperazineethanesulfonic acid (HEPES, #HEP-B) and RPMI 1640 (#RPMI-A) were from Capricorn scientific (Ebsdorfergrund, Germany), Eagle’s Minimum Essential Medium (EMEM, #P04-09500), Dulbecco’s Modified Eagle Medium (DMEM, #P04-04510), non-essential amino acids (NEAA, #P08-32100) and sodium pyruvate (#P04-43100) were from PAN-Biotech (Aidenbach, Germany), and ethylenediaminetetraacetic acid (EDTA, #A3145) was from PanReac AppliChem (Barcelona, Spain).

### 2.2. Mice

C57BL/6 wild-type (WT) mice were obtained from Jackson and expanded in our animal facility. The optineurin truncation mouse model that lacks the ubiquitin-binding domain (Optn^470T^) was generated as previously reported [23]. The Optn^470T^ mice used in this study were backcrossed to C57BL/6 genetic background 11 times; heterozygous mice were then crossed among themselves to obtain the homozygous Optn^470T/470T^ mice, henceforth called Optn^470T^ for simplicity. Male mice were used throughout the study. Animal procedures were performed according to the European Communities Council Directive of 24 November 1986 (86/609/EEC) and approved by the Ethics Committees of the Department of Biotechnology and Medical School of the University of Rijeka and the Ministry of Agriculture of the Republic of Croatia. Homozygous Optn^470T^ mice and control WT mice were used to generate primary myeloid cells, as described below.

### 2.3. Cell Lines

Neuro2A (N2A) mouse neuroblastoma cell line was cultured in Eagle’s Minimum Essential Medium (EMEM), and L929 mouse fibroblast cell line in Dulbecco’s Modified Eagle Medium (DMEM) supplemented with 10% foetal bovine serum (FBS), 2 mM L-glutamine, antibiotic/antimycotic solution (10,000 U/mL Penicillin, 10 mg/mL Streptomycin, 25 µg/mL Amphotericin B), and 10 mM HEPES, hereby referred to as complete DMEM. Complete EMEM was prepared in the same manner as complete DMEM, with an addition of 1× non-essential amino acids and 1 mM of sodium pyruvate. L929 cell line supernatant, which contains macrophage colony-stimulating factor (M-CSF), was collected 10 days after the cells reached complete confluence and was kept frozen until use. N2A and L929 cell lines were kind gifts from Dr. Stefano Campaner. 

### 2.4. Isolation and Cultivation of Primary Microglia and Bone Marrow-Derived Macrophages

To generate bone marrow-derived macrophages (BMDMs), femurs and tibias of 3-month-old and 2-year-old WT and Optn^470T^ mice were flushed using a 3 mL syringe with a 25G needle in RPMI 1640 medium supplemented with 10% FBS, 2 mM L-glutamine, antibiotic/antimycotic solution (10,000 U/mL Penicillin, 10 mg/mL Streptomycin, 25 µg/mL Amphotericin B), and 10 mM HEPES, referred to as complete RMPI 1640. The flushed bone marrow was filtered through a 70 μm cell strainer, centrifuged at 300× *g* for 5 min, and resuspended in complete RPMI 1640 medium enriched with 30% L929 cell line supernatant, as a source of M-CSF [36]. Bone marrow was cryopreserved at −80 °C in 10% DMSO/90% FBS. It was thawed at 37 °C, and BMDMs were differentiated during 5 days in culture prior to staining with PKH26 or CellTrace Far Red (described below) for phagocytosis experiments. Primary microglia were isolated from the brains of WT and Optn^470T^ neonatal pups (0–3 days postnatally), as previously described [25]. In brief, meninges were removed from the brains to prevent contamination with peripheral macrophages. Olfactory bulb and cerebellum were eliminated. Residual brain tissue was cut into small pieces, then incubated with 0.125% trypsin at 37 °C with 5% CO_2_. After 15 min, the trypsinization reaction was stopped with complete DMEM, and tissue was further triturated in the presence of DNase I (625 μg/mL). The resulting (mixed) cell suspension was filtered through 70 µm cell strainers and centrifuged at 110× *g* for 5 min. The cell pellets were resuspended into complete DMEM and plated onto 0.1 mg/mL poly-L-lysine-coated flasks. The medium was changed after 24 h and subsequently every 2–3 days until co-cultures reached full confluence (~7–10 days). Microglia were detached from the astrocyte layer by shaking for 16 h at 120 rpm and additional 4 h at 300 rpm. The microglia suspension was collected, centrifuged at 300× *g* for 5 min, stained with CellTrace Far Red (described below) and seeded onto poly-L-lysine-coated plates 48 h prior to experiments. The microglia were always used fresh (without freezing). 

### 2.5. Phagocytosis Assay

**Figure 1:**
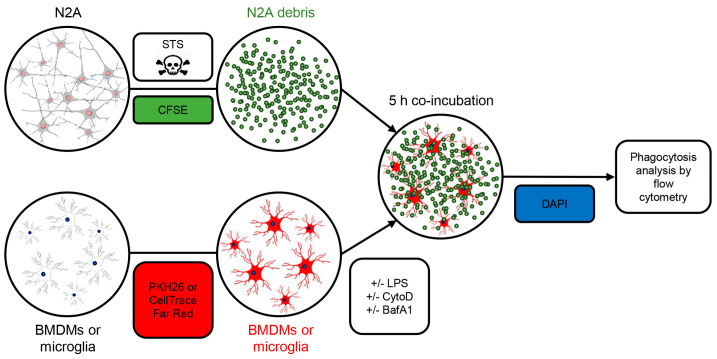
Setup for apoptotic cell phagocytosis assay. The Neuro2A (N2A) cells were treated with 2 μM staurosporine (STS) for 24 h to cause apoptotic cell death. The N2A debris was frozen and stained with 10 μM CFSE on the day of the experiment. BMDMs or neonatal microglia were labelled with PKH26 or CellTrace Far Red. When indicated, cells were pretreated with LPS and cytochalasin D (CytoD) to cause inflammation and block phagocytosis, respectively. Lysosomal inhibitor bafilomycin A1 (BafA1) was added 4 h prior to cell harvesting. CFSE-stained N2A debris was incubated with BMDMs or primary microglia for 5 h. The percentage of phagocytosing cells and the amount of phagocytosed material was assessed by flow cytometry. The dead cells were excluded by DAPI positivity.

For the phagocytosis assay, BMDMs were labelled with PKH26 or CellTrace Far Red, and microglia were labelled with CellTrace Far Red, following the manufacturer’s guidelines. This step was introduced to unambiguously distinguish the debris inside phagocytes from the extracellular debris. Briefly, 3 × 10^6^ BMDMs were washed twice in RPMI medium, stained in the dark with PKH26 (1:100 in phosphate-buffered saline (PBS)) for 3 min at room temperature or with CellTrace Far Red (1:5000 in PBS) for 20 min at +37 °C. Unincorporated dye was neutralized with complete RPMI medium. Microglia were washed once in PBS before staining with CellTrace Far Red. The dye was neutralized with 5 mL of complete DMEM, washed once more with complete medium, and centrifuged at 300× *g* for 5 min. Then, BMDMs and microglia were seeded in a 24-well plate at a density of 100,000 cells/well. We used apoptotic N2A cell debris or *E. coli* bioparticles as phagocytic targets. To obtain the apoptotic cell debris for the phagocytosis experiment, N2A cells were treated for 24 h with 2 μM staurosporine (STS), an apoptosis-inducing bacterial toxin. After the treatment, cellular debris was collected, washed once in PBS and frozen at −80 °C. On the day of the phagocytosis experiment, the N2A debris was thawed and labelled with 10 μM CFSE for 20 min at room temperature in the dark, following the manufacturer’s protocol. For the phagocytosis assay, the CFSE-labelled N2A debris was washed and added to the BMDM or microglial cultures in a 3:1 ratio. The plate was centrifuged (5 min at 200× *g*) to spin down the N2A fragments onto the cells, incubated for 5 h, then prepared for the flow cytometry. For bacterial phagocytosis, BMDMs were incubated with *E. coli* bioparticles (250 µg/mL in PBS) for 2 h and 5 h, respectively. To induce a proinflammatory phenotype, BMDMs and microglia were treated with 500 ng/mL of LPS for 24 h prior to the phagocytosis assay. Cytochalasin D (CytoD), an actin polymerization inhibitor that blocks phagocytosis, was used as a control to ensure that the detected CFSE signal was indeed actively phagocytosed material and not fragments trapped onto the cell surface. CytoD was added at 2 μM concentration 30 min before the cells were mixed with the N2A debris. Bafilomycin A1 (BafA1), an inhibitor of vacuolar H^+^ ATPase was added at 200 nM for 4 h before sample collection to prevent lysosomal degradation of phagocytosed material. The experimental setup for N2A phagocytosis is schematically summarized in Figure 1. Of note, during our phagocytosis setup we compared freshly prepared and previously frozen N2A debris with apoptotic cortical mouse neurons, obtained by the previously described method [37]. The highest level of phagocytosis was obtained upon incubation with frozen N2A debris; thus, this method was used for further experiments (Supplementary Figure S1).

### 2.6. Flow Cytometry

BMDM or microglia incubated with N2A debris were lifted from the plate using 10 mM EDTA for 15 min and washed twice in FACS flow medium (PBS with 2% FBS and 0.05% sodium azide). DAPI labelling of dead cells was done for 5 min at 300 nM concentration (at room temperature, in the dark), cells were subsequently washed, and resuspended in 200 μL of FACS flow medium. The percentage of phagocytic cells and the amount of phagocytosed material, measured as mean fluorescence intensity (MFI), was analysed on BD FACSAria III (BD Biosciences) flow cytometer, and the data were processed by FlowJo software version 10 (Tree Star) and FCS Express version 7 (De Novo Software). 

### 2.7. ELISA

Supernatants collected from phagocytosis experiments were assayed by enzyme-linked immunosorbent assay (ELISA). TNF-α and IL-10 cytokines were analysed Invitrogen and R&D Systems kits, respectively, according to manufacturer’s instructions. IFN-β was analysed by the previously published protocol [38], with slight modifications. In brief, 96-well plates were coated with capture antibody overnight at +4 °C (for TNF-α and IFN−β), or at room temperature (for IL-10). The next day, the plate was washed with washing buffer, and blocked with blocking buffer for 1 h at room temperature. After washing, standards and supernatants were added to the plate and incubated for 2 h at room temperature. Unstimulated and LPS-stimulated samples for IFN-β assay were analysed without dilution, whereas LPS-stimulated samples for TNF-α and IL-10 assays were diluted (1:5 to 1:50). The plates were then washed and subsequently incubated with biotinylated detection antibodies, washed again, and incubated with avidin-HRP conjugate for 20–30 min (for TNF-α and IL-10), or an HRP-labelled detection antibody for 1 h (for IFN-β). After the plate was washed, TMB substrate was added, and the reaction was stopped with 8.5% H_3_PO_4_ after 15–20 min. The absorbance was read on UV/Vis spectrophotometer at 450 nm.

### 2.8. Immunofluorescence Analysis

For immunofluorescence analysis of astrocyte-microglia co-cultures and purified microglia, the cells were plated on a poly-L-lysine treated chamber slides, fixed in paraformaldehyde for 15 min (4% in PBS), and subsequently permeabilized with 0.1% Triton X-100 in PBS for 15 min. Then, the cells were incubated in a blocking buffer (0.5% BSA in PBS) for 1 h at room temperature followed by incubation with anti-Iba1 (1:1000) and anti-CD68 (1:1000) primary antibodies overnight at +4 °C. After rinsing three times with PBS, cells were incubated with suitable secondary antibody (Alexa Fluor 488- or 546-conjugated, diluted 1:1000) for 1 h at room temperature. The nuclei were stained with 300 nM DAPI, and cells were mounted on glass slides with the Aqua-Poly/Mount mounting medium. Cells were imaged using Olympus IX83 microscope at 20× magnification. 

### 2.9. Statistical Analysis

The results were analysed with GraphPad Prism software version 8. An unpaired Student’s *t*-test was used to determine the *p*-value, and the values from 0.05 and less were considered statistically significant. Nonsignificant results were not marked.

## 3. Results

### 3.1. Optineurin Function Is Dispensable for Phagocytosis of N2A Debris in BMDMs Derived from Young Adult Mice

**Figure 2:**
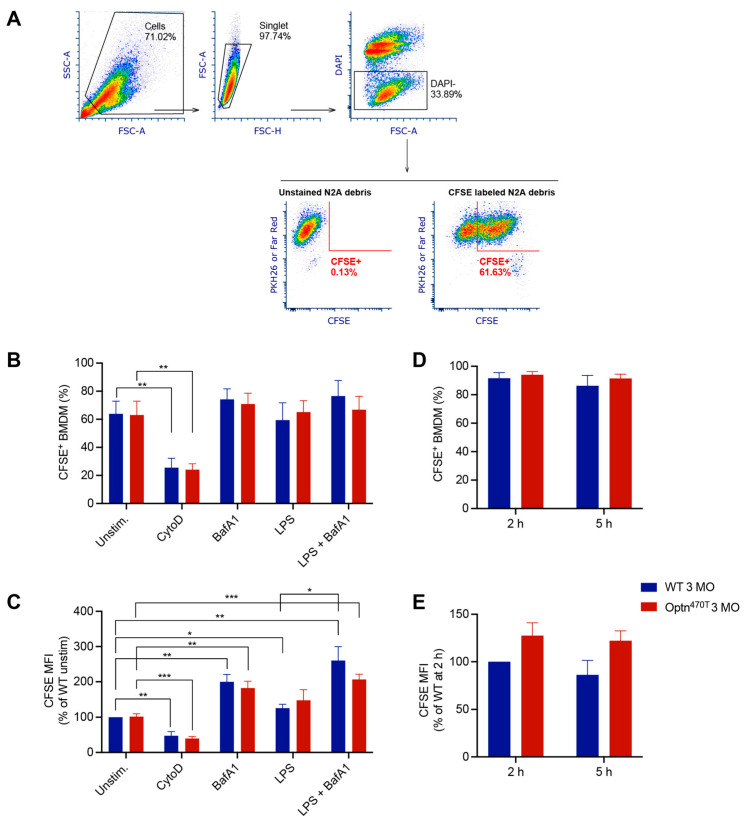
Optineurin insufficiency did not affect phagocytosis in young BMDMs. (**A**) Phagocytosis assay gating strategy: the main cell population was gated on FCS and SSC; cell aggregates were excluded by using FSC-A and FSC-H; live cells were gated as DAPI negative (remark: DAPI^+^ events included unphagocytosed N2A debris); BMDMs or microglia were detected as PKH26 or Far Red positive; the percent of phagocytic cells and the amount of phagocytosed CFSE^+^ N2A debris was quantified. (**B**) The percentage of phagocytic cells was determined for BMDMs from 3-month-old (3 MO) WT and Optn^470T^ mice that were incubated with CFSE-labelled N2A debris for 5 h. When indicated, BMDMs were pretreated with 2 µM CytoD for 30 min, treated with 200 nM BafA1 for 4 h before collection, and pretreated with 500 ng/mL of LPS for 24 h. (**C**) The amount of phagocytosed material was detected as MFI (normalized to WT unstimulated cells). For B-C the data are shown as mean ± SEM of 4–5 independent experiments. (**D**,**E**) BMDMs were incubated with *E. coli* bioparticles for the indicated times. (**D**) The percentage of cells that phagocytosed *E. coli* bioparticles and (**E**) MFI of phagocytosed material (normalised to WT phagocytosis at 2 h) are depicted for three individual experiments. Statistical analysis was done by unpaired Student’s *t*-test; * *p* < 0.05, ** *p* < 0.01, *** *p* < 0.001.

To evaluate if phagocytosis efficiency was dependent on functional optineurin, WT and optineurin-insufficient Optn^470T^ bone marrow-derived macrophages (BMDMs) were generated from young adult (3-month-old) mice and incubated with CFSE-labelled apoptotic N2A debris. The percentage of phagocytic (CFSE^+^) BMDMs was analysed by flow cytometry, and the phagocytosed N2A debris was quantified on a per cells basis (Figure 2A–C). Both WT and Optn^470T^ BMDMs demonstrated an active phagocytic uptake of the N2A debris, having a similar percentage of phagocytosing cells (~60%) (Figure 2B). CytoD, an actin inhibitor that blocks phagocytosis, was used as an internal control to prove that apoptotic N2A debris was indeed taken up by phagocytosis. CytoD treatment resulted in a 3-fold reduction in the percentage of phagocytosing cells, irrespectively of the genetic background, indicating that most of the N2A uptake occurred via phagosomes and not by unspecific binding to the cell surface and/or other means of cellular uptake. Notably, the mean fluorescence intensity (MFI) of phagocytosed N2A debris, which depicts phagocytosis on a per cell basis was also diminished upon CytoD treatment (Figure 2C). To evaluate putative differences in degradation of phagocytosed N2A debris between WT and Optn^470T^ BMDMs, which could potentially mask a different uptake between the genotypes, we blocked lysosomal degradation with Bafilomycin A1 (BafA1), an inhibitor of vacuolar H^+^ ATPase, necessary for phagosomal acidification and subsequent activation of lytic enzymes. Although BafA1 did not lead to increased percentage of phagocytic cells (Figure 2B), it resulted in an approximately 2-fold increase in MFI of phagocytosed material (Figure 2C), suggesting an active degradation. Nevertheless, BafA1 treatment did not uncover the differences between the genotypes, demonstrating a normal phagocytic uptake and phagosomal degradation in BMDMs lacking functional optineurin.

Having ruled out differences in the basal level of phagocytosis of apoptotic material between WT and Optn^470T^ BMDMs, we induced a proinflammatory phenotype by pretreatment with LPS for 24 h before the addition of the N2A debris. LPS alone or in combination with BafA1, compared to the corresponding untreated and BafA1 treated control BMDMs, did not modify the percentage of phagocytic WT and Optn^470T^ BMDMs (Figure 2B). However, LPS stimulation led to a small ~20–30% increase in MFI, reaching statistical significance for WT BMDMs, and showing a trend for Optn^470T^ BMDMs (Figure 2C). The combined treatment with LPS and BafA1 resulted in an approximately 2-fold increase in MFI of phagocytosed material (Figure 2C), as observed in unstimulated cells with BafA1, whereas a similar but nonsignificant trend has been observed in Optn^470T^ BMDMs. Overall, these results suggested that an inflammatory environment only mildly increased the rate of N2A debris uptake, with no substantial differences between the genotypes.

In our assays, we used neuroblastoma N2A cells as the closest proxy to dead neurons, which are a target for phagocytosis in neurodegenerative diseases. However, having seen that there was no difference between the genotypes in phagocytosis of apoptotic N2A cells in neither basal nor inflammatory conditions, we next evaluated phagocytosis of *E. coli* bioparticles, which have recently been used to show the differences in phagocytosis of monocyte-derived microglia-like cells from ALS and healthy subjects [34]. In our assay, *E. coli* bioparticles were efficiently phagocytosed by >80% of WT BMDMs from young adult mice at 2 h and 5 h of incubation (Figure 2D). Notably, it was needless to use CytoD control with pHrodo-labelled particles since this dye becomes fluorescent only upon phagosomal acidification, which makes it ideal for specific marking of the phagocytosed material. We also could not use BafA1 because inhibition of acidification would quench pH-sensitive pHrodo signal. Importantly, the percentage of active phagocytic cells and the amount of phagocytosed *E. coli* bioparticles were not different in Optn^470T^ BMDMs (Figure 2D and 2E). Thus, our results with *E. coli* were similar to those with N2A debris, with or without LPS pretreatment. Altogether, the data from both apoptotic cells and *E. coli* bioparticles showed that optineurin insufficiency had no role in the phagocytosis in BMDMs from young adult mice.

### 3.2. Optineurin Function was Dispensable for Phagocytosis of N2A Debris in BMDMs Derived from Aged Mice

**Figure 3:**
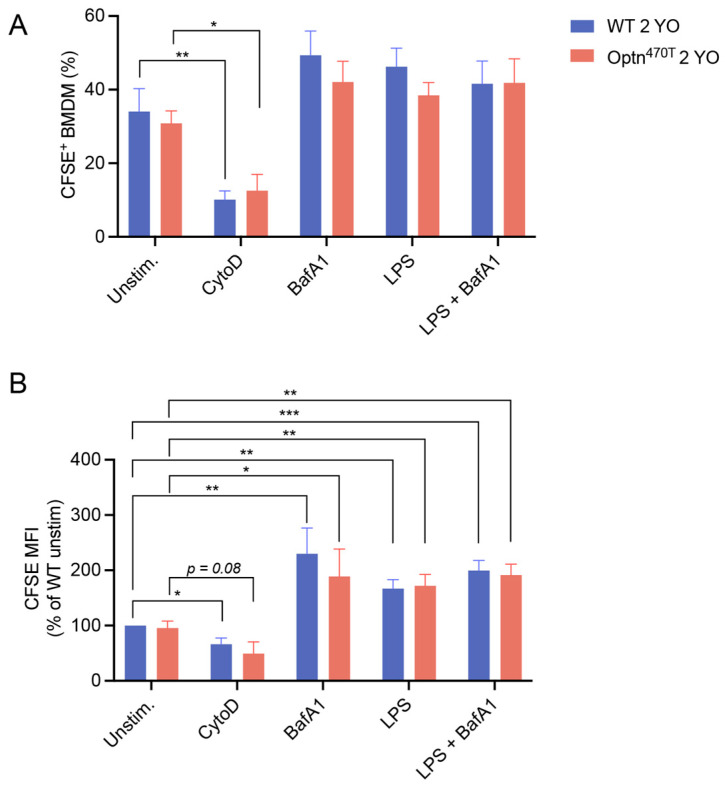
LPS pretreatment increased accumulation of phagocytic material in aged BMDMs, but this was unaffected by optineurin insufficiency. BMDMs from 2-year-old (2 YO) WT and Optn^470T^ mice were stained with PKH26 and incubated with CFSE-labelled N2A debris for 5 h. When indicated, BMDMs were pretreated with 2 µM CytoD for 30 min, treated with 200 nM BafA1 for 4 h before collection, and pretreated with 500 ng/mL of LPS for 24 h. Phagocytosis was assayed by flow cytometry and (**A**) percentage of phagocytic cells, and (**B**) CFSE MFI (normalized to WT unstimulated cells) were determined. The data are shown as mean ± SEM of 6 independent experiments, except for CytoD and BafA1 conditions, where they are shown for 3–4 independent experiments. Statistical analysis was done by unpaired Student’s *t*-test; * *p* < 0.05, ** *p* < 0.01, *** *p* < 0.001.

After showing that optineurin insufficiency had no effect on phagocytosis in BMDMs from young mice, we investigated whether the function of BMDMs from Optn^470T^ mice might be compromised during ageing, which is linked to various immune cell dysfunctions. To this end, we compared the phagocytic capacity of WT versus Optn^470T^ BMDMs derived from 2-year-old mice. We saw that a comparable fraction of WT and Optn^470T^ BMDMs was positive for the N2A debris (~35%). As expected, the phagocytic uptake upon CytoD treatment substantially decreased in both genotypes (Figure 3A,B). Similar to BMDMs from young mice, an inhibition of lysosomal degradation by BafA1, resulted in a ~2-fold increase in the amount of phagocytosed material (MFI) (Figure 3B), but did not affect the percentage of phagocytic cells. LPS or BafA1 did not significantly change the proportion of phagocytic cells in any of the genotypes, analogously to the findings in BMDMs derived from young mice (Figure 3A). Notably though, while the LPS-induced increase in MFI was minor in BMDMs from young mice, we observed a substantial (~70%) increase in phagocytosed N2A debris in BMDMs from aged mice, which was similar in both genotypes (Figure 3B). Interestingly, these values did not increase upon BafA1 treatment suggesting the presence of a lysosomal degradation block in LPS-pretreated aged BMDMs of both genotypes. Altogether, optineurin insufficiency had no role in the phagocytosis of N2A debris by WT and Optn^470T^ BMDMs from 2-year-old mice. Notably though, in aged mice, LPS decreased lysosomal degradation of phagocytosed material at a single-cell level, equally in both genotypes.

### 3.3. Phagocytosis and Proinflammatory Polarization in WT and Optn^470T^ BMDMs Become Impaired with Age

**Figure 4:**
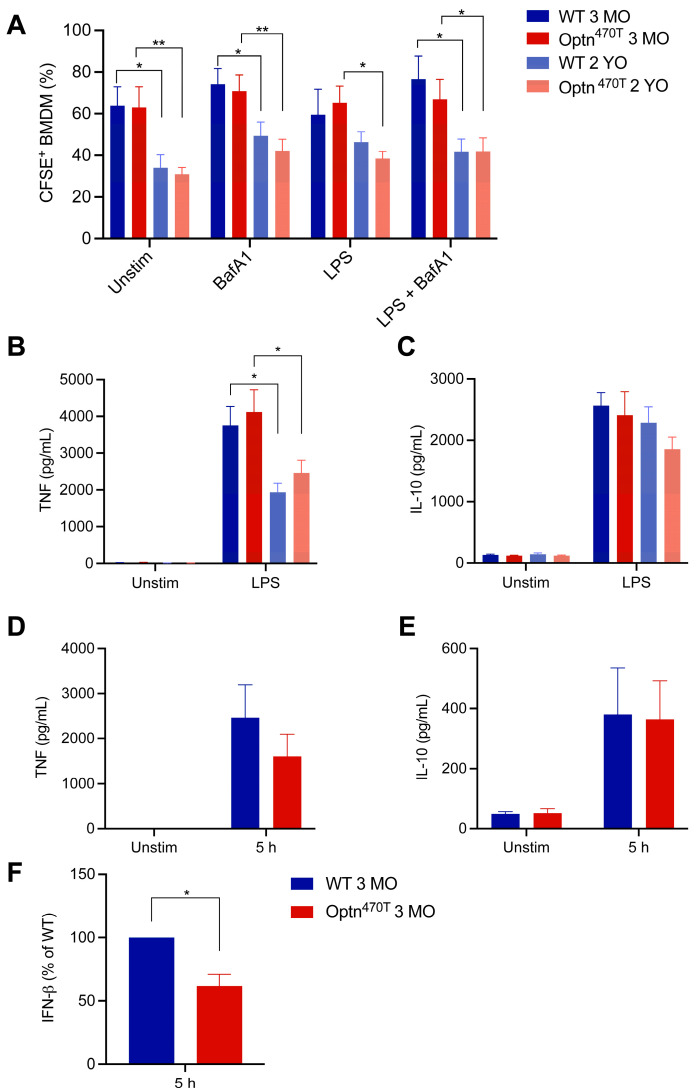
Ageing decreased the percentage of phagocytic BMDMs and pro-inflammatory cytokine secretion. (**A**) The percentage of phagocytic BMDMs from young (3-month-old; 3 MO) and aged mice (2-year-old; 2 YO), previously shown in Figure 2 and Figure 3, were depicted in the same graph. Supernatants from unstimulated and LPS-pretreated cells were assayed by ELISA for (**B**) TNF-α and (**C**) IL-10. The data are shown as mean ± SEM from 4–6 independent experiments ELISAs for (**D**) TNF-α, (**E**) IL-10, and (**F**) IFN-β were performed on supernatants from phagocytosis assays with *E. coli* bioparticles collected at 5 h. The data are shown as mean ± SEM from three independent experiments. For IFN-β, the data were normalized to WT in each of the three individual experiments. Statistical analysis was done by unpaired Student’s *t*-test; * *p* < 0.05, ** *p* < 0.01.

We noticed that phagocytic potential differed between young and old mice in our assays. Since this issue has remained controversial in the literature, we used this opportunity to analyse the effect of ageing on phagocytic capacity for N2A debris in BMDMs derived from young adult (3-month-old) and aged (2-year-old) mice. A statistically significant age-related drop in percentage of phagocytic cells (from ~60% to ~35%) was evident in both WT and Optn^470T^ BMDMs (Figure 4A). A similar difference between young and old BMDMs was preserved in both genotypes upon BafA1 treatment. LPS pretreatment showed a slightly smaller difference between BMDMs from young and aged mice, which reached significance only in Optn^470T^ while WT cells exhibited only a trend, perhaps due to a smaller number of repetitions than in other experiments (four). However, as shown above, LPS induced a bigger difference in MFI than it did in the percentage of phagocytic cells. Therefore, to gain more insight into LPS-induced differences between young and old mice on a per cell basis, these experiments should be performed in parallel, which was out of the scope of this study. To conclude, ageing led to substantially lower percentage of phagocytic cells.

To get more insight in BMDM functionality during ageing, we measured the amount of TNF-α and IL-10 cytokines released upon LPS stimulation, which are markers for proinflammatory and anti-inflammatory phenotype, respectively. TNF-α was undetectable in unstimulated BMDM cultures incubated together with N2A debris (Figure 4B). This corroborated previous findings that phagocytosis of apoptotic cells suppresses production of proinflammatory cytokines [35]. In contrast, LPS pretreatment led to an efficient TNF-α production in BMDMs incubated with N2A debris, which reached a comparable level in WT and Optn^470T^ cultures. Notably, a ~50% lower level of TNF-α was detected in cultures from BMDMs generated from aged mice, although there was still no difference between the genotypes. IL-10 was produced at low levels in unstimulated cells incubated with N2A debris, but its levels increased ~20-fold upon LPS pretreatment (Figure 4C). However, no statistically significant differences were present between young and aged mice. All together these results demonstrated that ageing suppressed the proinflammatory phenotype and decreased the phagocytic capacity, but optineurin did not affect any of these processes. 

Due to the reported regulation of IFN-β secretion by optineurin [19,23,39], and the fact that IFN-β has been shown to affect phagocytosis [40], we also evaluated the supernatants for this cytokine. However, the detected levels of IFN-β in co-cultures with apoptotic cells upon LPS pretreatment were very low, and even undetectable in some cases (data not shown), likely due to the fact that IFN-β is an early response cytokine, and the total duration of our assay was 29 h (24 h pretreatment + 5 h co-incubation with apoptotic N2A debris). Therefore, to validate our IFN-β and other cytokine assays, we evaluated the supernatants of co-cultures of young BMDMs with *E. coli* bioparticles. We detected TNF-α and IL-10 secretion upon 5 h, which was smaller than in LPS pretreatment experiment, which was expected as these cytokines peak later. Notably, at that early time point, we consistently saw IFN-β production upon *E. coli* bioparticle addition (Figure 4F). As previously observed [19,23,39], IFN-β was secreted at a lower level in Optn^470T^ than in WT cells. Therefore, IFN-β levels were lower in Optn^470T^ cells, but this did not affect phagocytosis of *E. coli* bioparticles. 

### 3.4. Optineurin Insufficiency Did Not Modify Phagocytosis of N2A Debris in Primary Microglia

**Figure 5:**
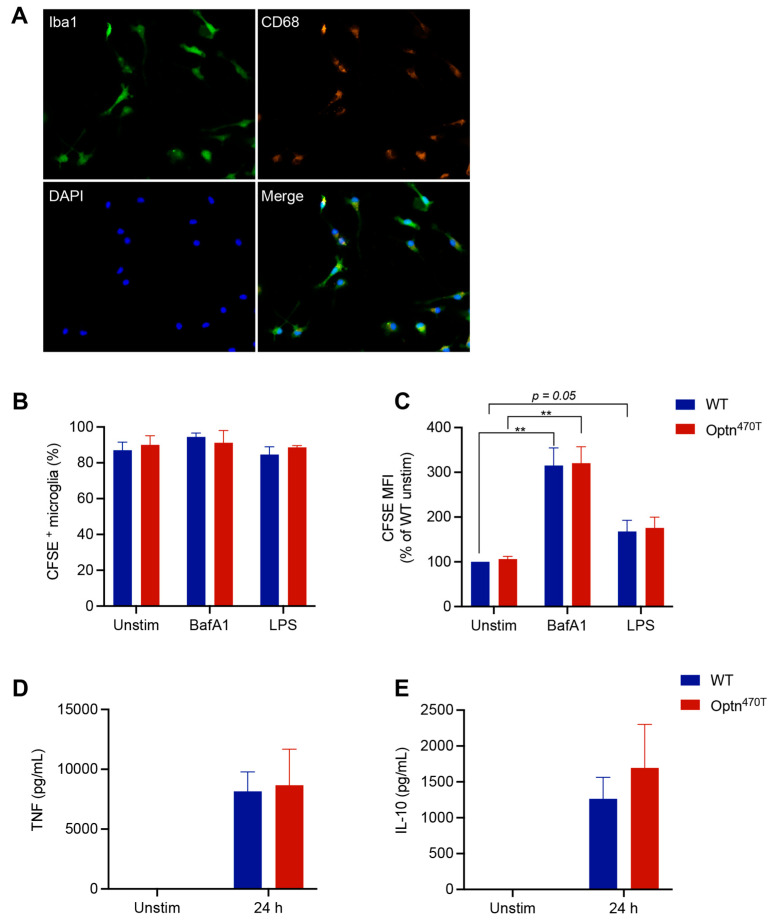
Optineurin function was dispensable for phagocytosis in primary microglia. Primary neonatal microglia were generated by co-culture with astrocytes. (**A**) Successful isolation and purity of WT microglia upon detachment from the astrocyte layer was assessed by immunofluorescence staining for Iba1 (green), CD68 (orange), and DAPI (blue). Representative single colour and merged images are shown. Microglia was labelled with CellTrace Far Red dye and incubated with CFSE-labelled N2A debris for 5 h. When indicated, BMDMs were pretreated with 500 ng/mL of LPS for 24 h or treated with 200 nM BafA1 for 4 h before collection (remark: due to paucity of primary microglia generated per pup, we limited our experiments to these selected conditions). Phagocytosis was assayed by flow cytometry and (**B**) percentage of phagocytic cells, and (**C**) CFSE MFI (normalized to WT unstimulated cells) was determined. Supernatants from unstimulated and LPS-pretreated microglia were assayed by ELISA for (**D**) TNF-α and (**E**) IL-10. The data are shown as mean ± SEM of two to four independent experiments. Statistical analysis was done by unpaired Student’s *t*-test; ** *p* < 0.01.

Microglia are the primary innate immune cells implicated in ALS, whose activation starts already in the pre-symptomatic phase of disease [41]. Therefore, to further investigate the effect of optineurin on phagocytosis in primary cells, we isolated purified Iba^+^ CD68^+^ microglia from neonatal mice (Figure 5A), incubated them with CFSE-labelled N2A debris, and analysed phagocytosis by flow cytometry. Both WT and Optn^470T^ microglia showed a comparable percentage of cells (~90%) that phagocytosed the N2A debris, and neither BafA1 nor LPS affected this percentage in any of the genotypes (Figure 5B). At the single cell level, BafA1-mediated lysosomal blockade led to a 3-fold increase in MFI of the internalized N2A debris (Figure 5C), suggesting that a substantial portion of N2A debris degrades via lysosomes over the course of the experiment in microglia. LPS showed a slight trend of higher MFI in both genotypes, but this did not reach a statistical significance, likely due to low number of repetitions (2–4, because of the paucity of microglia). Importantly, no differences were found between the WT and Optn^470T^ microglia in any of the experimental conditions (Figure 5B,C). To further probe the functionality of the primary isolated microglia, we measured the amount of TNF-α and IL-10 released in the cell culture medium upon LPS stimulation. Both TNF-α and IL-10 were undetectable in unstimulated co-cultures of apoptotic cells and microglia, but they were strongly produced upon LPS pretreatment (Figure 5D,E). Both cytokines were produced at a comparable level in WT and Optn^470T^ microglia, in line with the phagocytosis data. Overall, similar to our findings in BMDMs, these results showed that optineurin did not affect phagocytosis in primary microglia. 

## 4. Discussion

The central role of phagocytosis in neurodegenerative diseases has been recognized since the beginning of the 20th century when phagocytic microglia were identified surrounding amyloid-β plaques in Alzheimer’s disease [42]. However, their function in both disease promotion and suppression has been debated ever since due to the difficulties in monitoring phagocytosis, especially in vivo. For example, it has been reported that phagocytic microglia in rats carrying ALS-linked mutations surround motor neurons already at the presymptomatic stage of the disease (i.e., preceding neuronal loss) [32], but microglial activation markers, such as CD11b and MHC class II upregulation, and TNF-α and MCP-1/CCL2 positivity, were used as surrogate markers of phagocytosis without formally probing the phagocytic function of microglia. Some progress has been made with novel methods of direct analysis of phagocytosis (bacteria, bioparticles, and dead cells) in vitro, and, more recently, in vivo with stereotactic application of similar materials directly into selected brains regions of animals, which is technically challenging. A recent study has managed to demonstrate defective phagocytosis of *E. coli* bioparticles in microglia-like cells from sALS patients, which correlated with disease progression [34], proposing this as a common disease mechanism in a heterogenous population of ALS patients with different genetic and environmental makeup. To evaluate if phagocytosis is impaired in a model of optineurin insufficiency, we analysed phagocytosis in primary macrophages and microglia from Optn^470T^ mice. 

We first evaluated unmanipulated cells from young adult animals and found no difference in the percentage of phagocytic cells or the amount of phagocytosed apoptotic N2A debris in WT and Optn^470T^ BMDMs and neonatal microglia. We also applied BafA1 to block lysosomal degradation, which expectedly resulted in increased accumulation of phagocytosed N2A debris per cell, but did not uncover differences between the genotypes. Thus, optineurin insufficiency did not diminish phagocytosis at steady state. Notably, no TNF-α was secreted if BMDMs or microglia were incubated with apoptotic cells alone, corroborating previous notion that apoptotic cell removal is not an inflammatory stimulus [35]. Next, we evaluated if proinflammatory stimulation by LPS, a commonly used TLR agonist that simultaneously triggers NF-κB and TBK1 pathway activation, would trigger a phagocytosis defect in Optn^470T^ cells. This is important because inflammation in the CNS is one of the key drivers of neurodegenerative processes. An earlier study has shown that the uptake and degradation of phagocytosed apoptotic pheochromocytoma 12 cells by primary neonatal microglia increased upon their activation with LPS and TNF-α [43]. We also observed a slight ~20% increase in the uptake of apoptotic N2A debris upon LPS treatment in WT BMDMs, and a trend of similarly increased uptake in microglia, but this occurred without a significant increase in the percentage of phagocytic cells. Notably, optineurin insufficiency did not further affect the phagocytic potential. Surprised with this finding, we also compared phagocytosis of *E. coli* bioparticles in WT and Optn^470T^ BMDMs. *E. coli* bioparticles are naturally LPS-laden, so unlike apoptotic cells, which are known to suppress inflammation, they should be directly proinflammatory. Nevertheless, we observed no differences in *E. coli* bioparticle phagocytosis between WT and Optn^470T^ BMDMs. In contrast to phagocytosis without LPS pretreatment, which resulted in no TNF-α and only modest IL-10 production, LPS pretreatment led to a strong TNF-α and IL-10 production in both BMDMs and microglia, which was indistinguishable between the genotypes. Similarly, *E. coli* bioparticle phagocytosis also led to equivalent cytokine production in WT and Optn^470T^ BMDMs. Thus, optineurin insufficiency did not affect phagocytosis and accompanying cytokine secretion even when the cells were exposed to an inflammatory environment. 

We also evaluated the effect of ageing on phagocytosis. Ageing, as the most prominent risk factor for neurodegenerative diseases, is linked to increased myelin fragmentation [44], coupled with a decline in microglial functions [30]. Ageing affects several steps in phagocytosis of protein aggregates and/or cellular debris including recognition of phagocytic material (eat-me versus do-not-eat-me signals), its lysosomal degradation and ensuing oxidative stress [45,46,47,48]. Therefore, aged microglia can become overloaded with undigested products (myelin, damaged mitochondria, aggregated proteins, and others), in part visible as lipofuscin. This in turn suppresses their protective functions and affects their inflammatory secretion profiles. It is interesting though, that both an increase and decrease in phagocytosis and hyper- and hypo-active microglia have been linked to ALS and other neurodegenerative diseases, often differently in different experimental system and/or at distinct stages of disease progression. The same was reported for macrophages. For example, thioglycolate-elicited peritoneal macrophages isolated from young and old mice were equally efficient in phagocytosis of *Porphyromonas gingivalis*, a common periodontal pathogen [49]. In contrast, in situ phagocytosis of UV-killed keratinocytes and apoptotic Jurkat cells injected into peritoneal cavity were less efficient in aged mice [46]. Here, we compared phagocytosis of apoptotic N2A debris by BMDMs derived from young adult (3-month-old) and aged (2-year-old) mice. Strikingly, we found that ~50% fewer WT BMDMs generated from aged mice were phagocytic when compared to those generated from young adult mice. LPS pretreatment in the aged mice led to a stronger accumulation of phagocytosed material, and our BafA1 experiments showed that this resulted from decreased degradation rather than increased uptake of N2A debris. However, ageing did not uncover differences between Optn^470T^ and WT mice in any of the parameters evaluated. As observed in BMDMs from young mice, no TNF-α was secreted when aged BMDMs were incubated with apoptotic cells alone. This argued that aged BMDMs did not exhibit an enhanced basal activation. When BMDMs were pretreated with LPS, an efficient TNF-α secretion was observed in BMDMs from young mice, whereas BMDMs from aged mice secreted ~2-fold less TNF-α. Some IL-10 was detected during co-incubation with apoptotic N2A debris even without pretreatment, but the secretion increased >20-fold upon LPS pretreatment. However, there was no difference in IL-10 secretion in BMDMs from young and aged mice. In conclusion, BMDMs from aged mice showed lower phagocytic potential, and lesser capacity for TNF-α secretion than those from young mice. Nevertheless, this ageing-induced decline was unaffected by optineurin insufficiency. 

It is unclear at this point why optineurin insufficiency proved to have no effect on phagocytosis in primary mouse myeloid cells, in contrast to recently reported sALS patients’ microglia [34]. Optineurin could have influenced phagocytosis by several direct and indirect mechanisms including modulating IFN-β and TDP-43 levels, or by influencing other facets of the immune response. Phagocytosis has been shown to be upregulated by IFN-β in multiple sclerosis lesions and demyelinated organotypic slices [40]. Since IFN-β production is dependent on activation of TBK1 by scaffolding function of optineurin [23,24,25,39], optineurin could have supported phagocytosis by upregulating IFN-β. However, IFN-β was not secreted in BMDM or microglia co-cultures with apoptotic N2A debris, and its levels were very low (or undetectable in some samples) upon 29 h of LPS pretreatment (data not shown), consistent with it being an early response cytokine. Notably though, we found decreased IFN-β in Optn^470T^ compared to WT BMDM cultures upon 5 h of co-incubation with *E. coli* bioparticles. Since this happened without any differences in phagocytosis, it is unlikely that IFN-β influenced phagocytosis in our assays. It has also been reported that TDP-43, a protein that aggregates in >95% of ALS patients regardless of their genetic background, regulates microglial phagocytosis: microglial TDP-43 depletion increased amyloid-β clearance both in vitro and in vivo [50]. Notably, Quek et al. showed that sALS patients’ microglia-like cells, which had defective phagocytosis, also exhibited TDP-43 and phosphorylated TDP-43 pathology [34]. We have recently shown that optineurin primary microglia and BMDMs from Optn^470T^ mice had increased TDP-43 protein levels [51], but without an overt TDP-43 pathology. This could perhaps explain the differences between mouse and human data in phagocytosis assays. Alternatively, mutations in the immune system regulating genes, could indeed act by a different mode of action in ALS. Notably, TBK1 haploinsufficiency had a biphasic effect on ALS progression in *SOD1* transgenic mice: it accelerated disease onset, but decelerated disease progression by suppressing inflammation [14]. It is possible that optineurin also has dual role in promoting immune-mediated dysfunction and healthy responses, depending on other environmental triggers. Notably, we only analysed the cell-intrinsic effects of optineurin here; thus, further studies should be performed to analyse other putative extrinsic triggers. It would also be interesting to see if other modes of cell death that are present in neurodegenerative diseases, such as inflammasome-mediated cell death (pyroptosis), would uncover differences in phagocytosis between Optn^470T^, and WT myeloid cells. Lastly, it is possible that some ALS mechanisms cannot be studied in a mouse model. For this reason, our future plans include analysis of optineurin functions in human samples.

## 5. Conclusions

To study the potential role of optineurin in phagocytosis, we set up a flow cytometry-based phagocytosis assays of apoptotic N2A debris and *E. coli* bioparticles in primary BMDMs and microglia from WT and Optn^470T^ mice. Optineurin could have potentially influenced phagocytosis by several direct and indirect mechanisms, including modulation of the IFN-β and TDP-43 levels, or by influencing other facets of the immune response in ALS patients [25,50,52,53,54]. However, we found no differences in the percentage of phagocytic cells and the amount of phagocytosed material in BMDMs and microglia from WT and Optn^470T^ mice. Inflammatory environment increased amount of phagocytosed N2A debris in both genotypes, especially in the BMDMs arising from aged mice. This was due to a decreased degradation rather than increased uptake of phagocytosed N2A debris. We also report that ageing substantially decreased the percentage of phagocytic BMDMs and the accompanying proinflammatory cytokine secretion. In contrast, ageing did not affect the number of phagocytic cells between the genotypes. In conclusion, our results show that optineurin insufficiency did not diminish phagocytosis in BMDMs and primary mouse microglia.

## Data Availability

Not applicable.

## Supplementary Materials

The following supporting information can be downloaded at: https://www.mdpi.com/article/10.3390/biology12020240/s1, Figure S1. Frozen N2A debris is superior to fresh N2A debris and frozen primary neurons in phagocytosis assays. WT and Optn^470T^ PKH26-stained BMDMs from 2-year-old mice were incubated with CFSE-stained debris from: (1) fresh N2A, (2) frozen N2A, and (3) primary cortical mouse neurons. (A) Percentage of phagocytic BMDMs and (B) the amount of phagocytosed material were determined upon 5 h incubation. Due to its superior phagocytosis, frozen N2A debris was chosen for subsequent analyses.
