# Supplementary figures and images for: Ageing-Induced Decline in Primary Myeloid Cell Phagocytosis Is Unaffected by Optineurin Insufficiency
Source: Biology (Basel). 2023 Feb 3;12(2):240. doi: 10.3390/biology12020240 (PMC9953198; doi:10.3390/biology12020240)

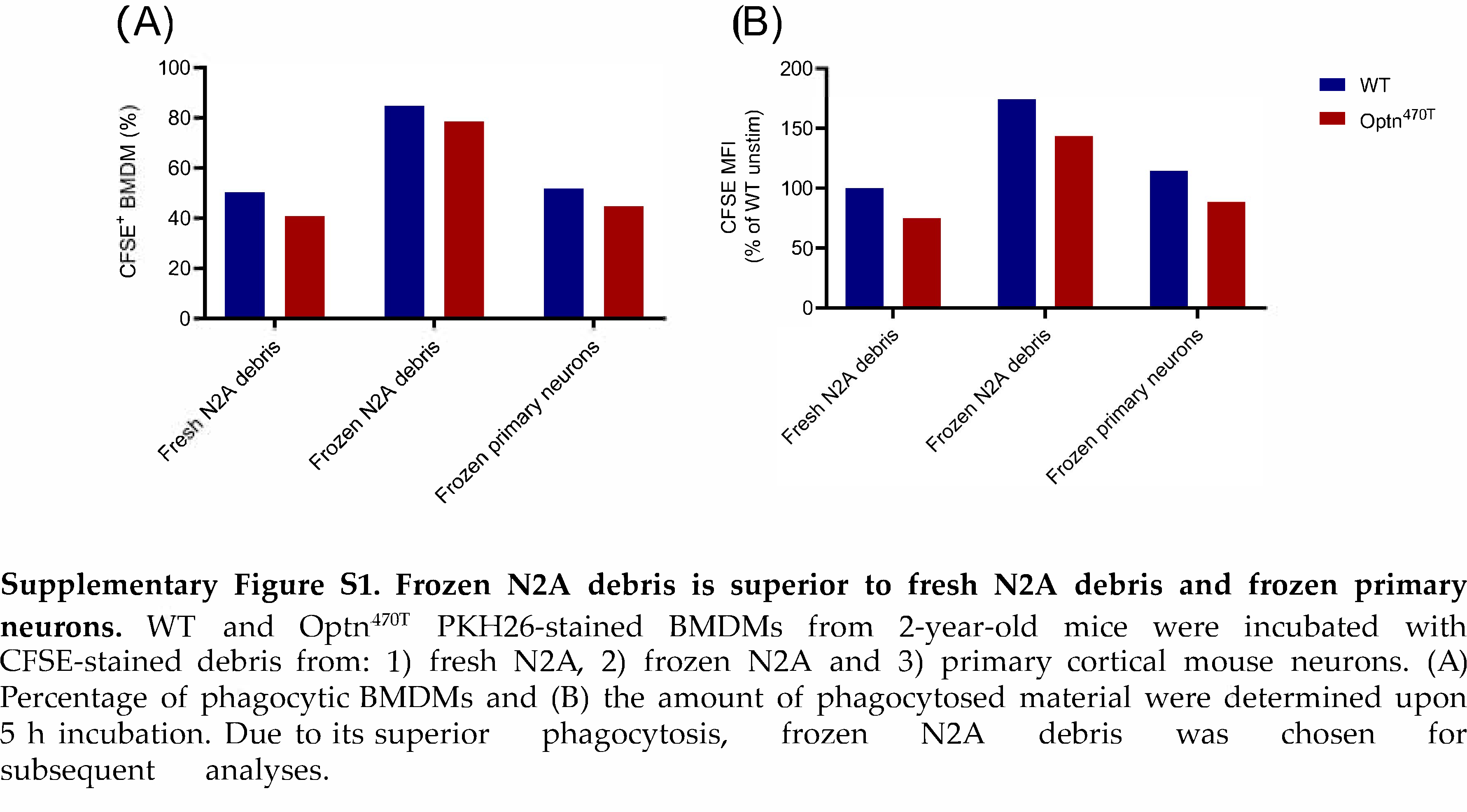

Supplement: Supplementary file 1 [file biology-12-00240-s001.zip › Figure S1.tif]
